# Serial blockface SEM suggests that stem cells may participate in adult notochord growth in an invertebrate chordate, the Bahamas lancelet

**DOI:** 10.1186/s13227-020-00167-6

**Published:** 2020-10-17

**Authors:** Nicholas D. Holland, Ildiko M. L. Somorjai

**Affiliations:** 1 Marine Biology Research Division, Scripps Institution of Oceanography, University of California At San Diego, La Jolla, CA 92093 USA; 2 School of Biology, University of Saint Andrews, St. Andrews, KY16 9ST Scotland

**Keywords:** Cephalochordata, Lancelet, Amphioxus, Serial blockface scanning electron microscopy (SBSEM), Notochord, Stem cell, Progenitor cell, Intervertebral disc, Nucleus pulposus

## Abstract

**Background:**

The cellular basis of adult growth in cephalochordates (lancelets or amphioxus) has received little attention. Lancelets and their constituent organs grow slowly but continuously during adult life. Here, we consider whether this slow organ growth involves tissue-specific stem cells. Specifically, we focus on the cell populations in the notochord of an adult lancelet and use serial blockface scanning electron microscopy (SBSEM) to reconstruct the three-dimensional fine structure of all the cells in a tissue volume considerably larger than normally imaged with this technique.

**Results:**

In the notochordal region studied, we identified 10 cells with stem cell-like morphology at the posterior tip of the organ, 160 progenitor (Müller) cells arranged along its surface, and 385 highly differentiated lamellar cells constituting its core. Each cell type could clearly be distinguished on the basis of cytoplasmic density and overall cell shape. Moreover, because of the large sample size, transitions between cell types were obvious.

**Conclusions:**

For the notochord of adult lancelets, a reasonable interpretation of our data indicates growth of the organ is based on stem cells that self-renew and also give rise to progenitor cells that, in turn, differentiate into lamellar cells. Our discussion compares the cellular basis of adult notochord growth among chordates in general. In the vertebrates, several studies implied that proliferating cells (chordoblasts) in the cortex of the organ might be stem cells. However, we think it is more likely that such cells actually constitute a progenitor population downstream from and maintained by inconspicuous stem cells. We venture to suggest that careful searches should find stem cells in the adult notochords of many vertebrates, although possibly not in the notochordal vestiges (nucleus pulposus regions) of mammals, where the presence of endogenous proliferating cells remains controversial.

## Background

The phylum Chordata consists of the basally branching cephalochordates (lancelets or amphioxus) as well as tunicates and vertebrates [1]. Because of this key phylogenetic position and a very slow rate of evolution [2–4], lancelets can give insights into the starting conditions for characters that continued evolving during the emergence of the vertebrates. One such character is the notochord, a defining feature of chordates [5]. In lancelets, the organ runs down the axis of the body and grows throughout life while maintaining a consistent histological organization. In contrast to many adult vertebrates, lancelets never subdivide the notochordal tissue with interpolated regions of cartilage and/or bone.

In the adult lancelet notochord, new cells are added to provide for its enlargement and also to replace terminally differentiated cells lost due to normal wear and tear. The cellular basis for this growth and maintenance was previously attributed to cortical (Müller) cells proliferating at the surface of the notochord and then differentiating into the lamellar cells making up the core of the organ [6–8]. These older studies date from a time when no distinction was made between stem cells and progenitor (also sometimes called transit amplifying) cells in solid tissue; both were simply considered together under names like *mitotic zone*, *DNA synthesis zone*, *proliferating cell population*, etc.

The purpose of the present study was to determine whether the adult lancelet notochord includes not only the proliferating Müller cells already known, but also an additional population of stem cells in the modern sense of the word. To accomplish this, it was necessary to study the fine structure of an exceptionally large sample of cells in situ. Therefore, we used serial blockface scanning electron microscopy (SBSEM), a technique yielding three-dimensional reconstructions at the resolution of transmission microscopy (TEM). By visualizing a much larger tissue volume than usual with this technique, we found a small cluster of likely stem cells at the posterior end of the notochord. They appear to give rise to the Müller cells that, in turn, transition to the differentiated lamellar cells. It is reasonable to assume that stem cell-based notochord growth was an ancestral chordate character that carried forward during vertebrate evolution, even as the organ tended to become increasingly vestigial. Ultimately, in adult mammals, the notochord persists as remnants (nucleus pulposus regions) in which the presence of any dividing cells is currently debated.

## Methods

### Animal collection and initial processing

**Fig. 1:**
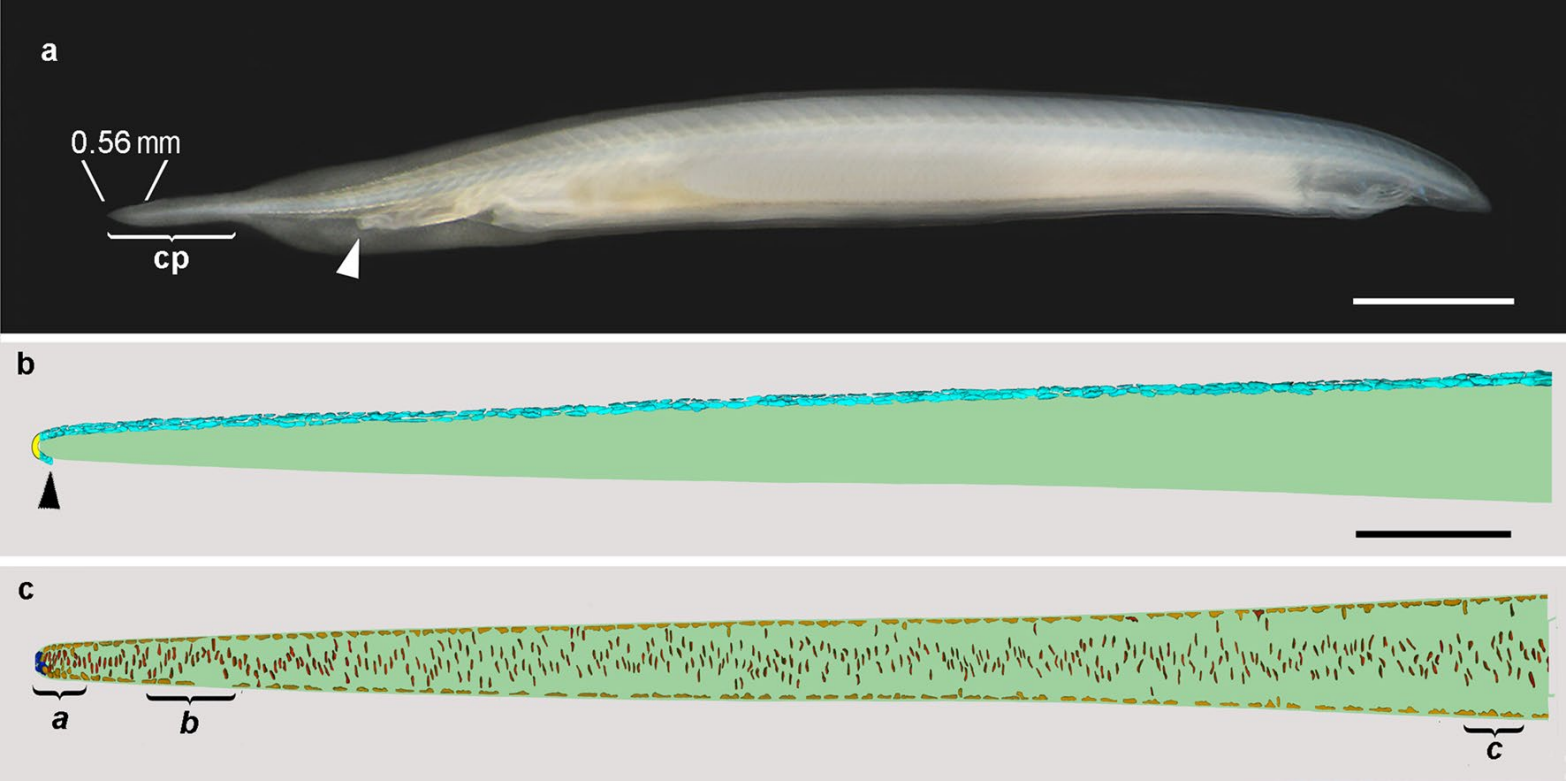
**a** Living adult Bahamas lancelet (*Asymmetron lucayanum*) with head toward right and the anus indicated by the arrowhead. The tail terminates as a slender caudal process (cp); the 0.56-mm region reconstructed by SBSEM is indicated. Scale bar = 2 mm. **b** Reconstructions of all the CNS cell nuclei (turquoise) in the notochord region studied. The CNS terminates (arrowhead) just ventral to notochordal tip. The yellow area indicates where about 20 CNS cells were trimmed awafy during specimen orientation. Scale bar (also applicable to **c**) = 50 μm. **c** Reconstructions of all the notochord cell nuclei in the region studied: putative stem cell nuclei are blue, Müller cell nuclei are dark yellow, and lamellar cell nuclei are red. Brackets *a*, *b*, and *c* indicate zones shown in detail in subsequent figures

Specimens of the Bahamas lancelet, *Asymmetron lucayanum* (Fig. 1), were sieved from the soft substratum at low tide in the Bimini lagoon, 25.72297°N, 79.29288 °W [9]. The animals studied here were adults 1.8 cm long, the most abundant size class collected. They were in a state of slow somatic growth: if fed in the laboratory, they reach a length to 2.2 cm in about 18 months before dying, evidently of old age. From six of the lancelets, the tail was cut off and processed for SBSEM. The present results are based on the most favorably oriented specimen, although ancillary observations were made on the others before poor alignment became evident and SBSEM was discontinued.

### Fixation, post-fixation, SBSEM, and three-dimensional reconstruction

Initial fixation was for 2 weeks at 4 °C in 0.15 M cacodylate buffer (pH 7.4) containing 2% formaldehyde, 1.5% glutaraldehyde, and 2 mM CaCl_2_ [10]. The samples were post-fixed successively in reduced osmium tetroxide, thiocarbohydrazide, osmium tetroxide, uranyl acetate, and lead aspartate under conditions specified in table 1 of reference [11]. After ethanol dehydration, the specimens were transferred through acetone and embedded in Durcupan resin. Blocks were oriented for cross-sectioning starting at the tail tip and proceeding anteriorly. The SBSEM was accomplished in a 3View system (Gatan, Pleasanton, CA) installed in a Zeiss Merlin SEM. After the microscope records an image of the blockface by backscattered electrons, a microtome in the specimen chamber shaves off a thin superficial layer from the face of the block, exposing a new surface to scan [12, 13]. The alternation of scanning and shaving generates uninterrupted serial images that look superficially like conventional TEM.

A layer 0.25 μm thick was shaved off the blockface between each scan. For the most favorably oriented specimen, we scanned 2,240 consecutive block faces, equal to a posterior-to-anterior distance of 0.56 mm. Although this appears quite modest in terms of the total length of the animal, it represents a much larger tissue volume than usual for contemporary SBSEM studies. The SBSEM image series was analyzed with *Reconstruct* software, which is available gratis from https://www.bu.edu/neural/Reconstruct.html [14, 15]. The reconstructed cells were visualized in three dimensions as continuous Boissonnat surfaces, sometimes rendered semitransparent to show the intracellular organelles.

### Statistical analysis

Mitochondrial number per cell and volume per cell within notochord lamellar cells were analyzed using the two-tailed Mann–Whitney* U* test (https://www.socscistatistics.com/). Parametric tests were deemed inappropriate due to small sample size, or because conditions of normality and homogeneity of variances were not met as assessed by Kolmogorov–Smirnov and Levene’s tests.

## Results

### General orientation

For the present fine-structural description of the notochord in an adult cephalochordate, the species studied was *Asymmetron lucayanum*, the Bahamas lancelet (Fig. 1a). The 0.56-mm length of notochord visualized with SBSEM is indicated in the figure.

### Central nervous system (CNS) associated with the notochord

The posterior region of the CNS is included here (Fig. 1b) because it is a close neighbor of the notochord and a likely candidate for providing at least part of the microenvironment influencing the maintenance and differentiation of notochordal stem cells. The CNS cells in the reconstructed region are mostly, and possibly entirely, neuroglia [16]. Low-magnification SBSEM could not resolve cytoplasmic boundaries between these cells, but their distribution is adequately shown by depictions of their nuclei alone. The CNS extends along the dorsal side of the notochord, curls ventrally around its tip, and terminates at the arrowhead in Fig. 1b. In the figure, there are 368 CNS nuclei dorsal to the notochord and 5 ventral to it; the intervening yellow zone is where about 20 of the most posterior CNS cells were lost when the block was trimmed for sectioning.

### Overview of notochord cells

The cephalochordate notochord extends the entire length of the body and is enclosed in a collagenous sheath about 0.3 μm thick [8]. Figure 1c shows the distribution of all the notochord cells in the region studied. For clarity, only the nuclei are illustrated (cytoplasmic boundaries are shown in most of the subsequent figures); brackets *a*, *b*, and *c* indicate three zones reconstructed, respectively, from 102, 149, and 110 consecutive SBSEM sections. For each zone, the cellular structures are considered in detail in the results below.

There are three major categories of cells in the notochord region shown in Fig. 1c. First are the putative stem cells (blue nuclei), 10 in all, clustered at the extreme posterior end of the notochord. Second are the Müller cells (dark yellow nuclei), which are named for their discoverer [17] (and should not be confused with vertebrate retinal glial cells with the same name). In the region studied, they extend along the surface of the notochord in a dorsal row of 80 and a ventral row of 69. In addition, 12 of them occur deeper in the notochord, especially just anterior to the proposed stem cells. The third cell type (red nuclei), we will call lamellar cells instead of their older designations as plate-like cells [18] or central cells [19]. These highly differentiated cells constitute the bulk of the notochord. In the region studied, 373 lamellar cells have nuclei positioned deep in the notochord, but the nuclei of 12 others occur superficially, at the level of the dorsal and ventral Müller cells.

The subsurface locations of some Müller cell nuclei and the superficial locations of some lamellar cell nuclei were interpreted over a century ago [6, 7] to indicate that the former cell type differentiates into the latter. We agree with this, but it is only part of a more complicated overall scheme of notochord cell production and differentiation. The likely beginning of the story, missing until now, is the small posterior group of putative stem cells already mentioned.

### Zone *a*: likely stem cells

**Fig. 2:**
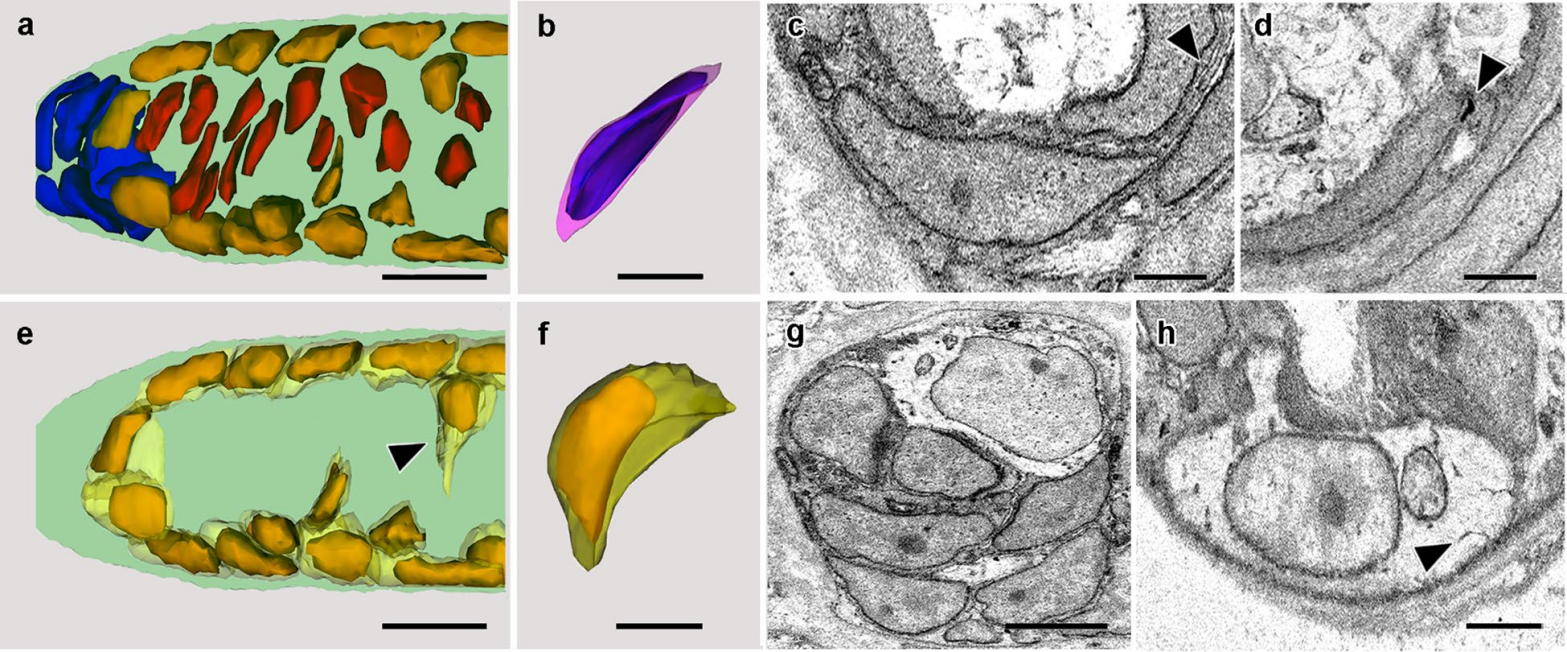
**a** Enlargement of zone *a* in Fig. 1c, showing putative stem cells (blue nuclei), Müller cells (dark yellow nuclei), and differentiated lamellar cells (red nuclei). Scale bar = 5 μm. **b** Putative stem cell (blue nucleus surrounded by pink cytoplasm) reconstructed in three dimensions to show its concavo-convex shape. Scale bar = 2 μm. **c** SBSEM image of stem cell; the cytoplasm includes a mitochondrion and profiles of rough endoplasmic reticulum (arrowhead). Scale bar = 1 μm. **d** Chromatoid body (arrowhead) adjacent to nucleus of putative stem cell. Scale bar = 1 μm. **e** Nucleus and cytoplasm of Müller cells in zone *a*; some of these (arrowhead) appear to be penetrating from the periphery into the core of the notochord. Scale bar = 5 μm. **f** One of the most posterior Müller cells in (**e**), turned to emphasize its concavo-convex shape. Scale bar = 2 μm. **g** SBSEM image showing the foregoing Müller cell (top right) with conspicuously lucent nucleus and cytoplasm. Scale bar = 2 μm. **h** SBSEM image showing a ventral Müller cell with a mitochondrion and deep invaginations (arrowhead) of the plasma membrane within the lucent cytoplasm; the cell extends a blunt cytoplasmic projection at top left. Scale bar = 1 μm

Figure 2a is an enlarged view of zone *a*. The posterior cluster of proposed stem cells (blue nuclei) is conspicuous in SBSEM. Figure 2b shows a 3-dimensional reconstruction of one of these cells oriented to emphasize that it is shaped like a thick concavo-convex lens. The fine structure of the same cell is shown in an SBSEM image (Fig. 2c). The relatively dense nucleus contains a nucleolus, and the dense, scanty cytoplasm strongly contrasts with the lucent cytoplasm of the other two kinds of notochordal cells. Several cytoplasmic organelles often found in stem cells are present: namely, a single mitochondrion, some free ribosomes, and inconspicuous profiles of rough endoplasmic reticulum. In addition, in the cytoplasm just outside the nucleus, there are several dense patches (Fig. 2d, arrowhead) resembling the protein- and RNA-rich structures called chromatoid bodies or nuage in somatic stem cells of other animals [20]. That said, it should be stressed that no combination of static cytological features suffices to identify stem cells unequivocally. A firm identification ultimately needs to be based on the details of their proliferation and cytodifferentiative fate.

### Zone *a*: Müller cells

Figure 2e shows the nucleus (dark yellow) and cytoplasm (light yellow) of each Müller cell in zone *a*. The two most posterior Müller cells are located deep within the notochord. One is illustrated in Fig. 2f to emphasize that, unlike its oval counterparts along the surface of the notochord, it is shaped like a concavo-convex lens. This distinctive shape indicates its recent production from the putative stem cells, from which it otherwise differs in the extreme in its voluminous and lucent cytoplasm (Fig. 2g, top right). The nucleus contains a nucleolus, the plasma membrane is smooth, and the cytoplasm includes a mitochondrion and several lysosomes.

The other Müller cells running in dorsal and ventral rows along the surface of the notochord have similarly lucent cytoplasm, but, as already mentioned, they are approximately oval (Fig. 2h), except where some extend short blunt cytoplasmic processes basally. One peculiarity of these superficial cells is that the plasma membrane is invaginated in several places to form narrow canaliculi in the cytoplasm (Fig. 2h, arrowhead). Similar canaliculi were previously observed in Müller cells of another species of cephalochordate [18], but their function is unknown. A few of the Müller cells (Fig. 2e, arrowhead) appear to be migrating from their superficial position (with a prominent cytoplasmic process in advance) toward the core of the notochord. Such cells, although as yet lacking cytoplasmic muscle fibers (introduced in the next section), are evidently on the verge of transitioning to lamellar cells.

### Zone *a*: lamellar cells

**Fig. 3:**
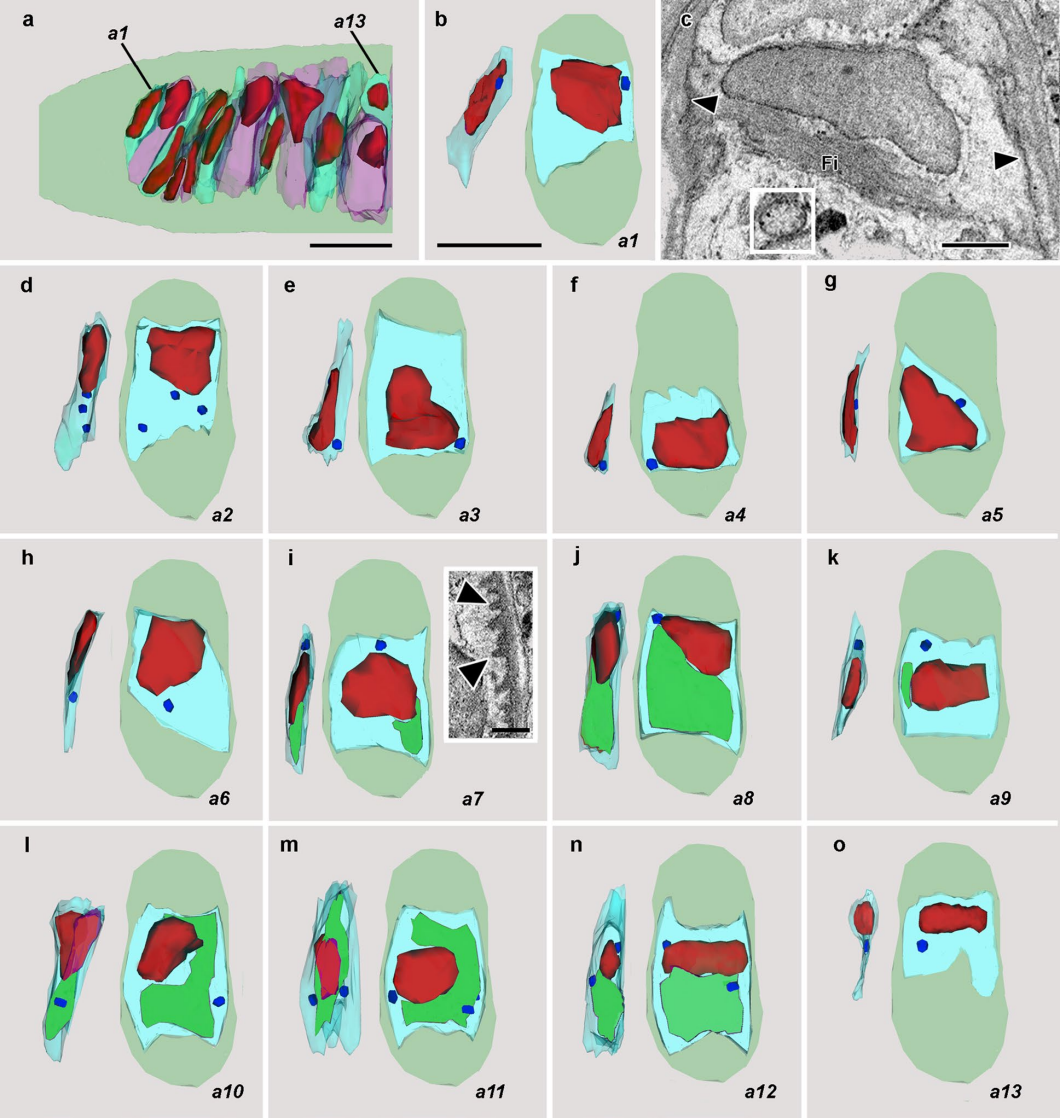
**a** Enlargement of zone *a* showing lamellar cells (with alternatively blue-green and pink cytoplasm to set the cells apart clearly) designated *a1* through *a13*. Scale bar = 5 μm. **b** Lamellar cell *a1* in side view at left and in posterior view at right against a light green silhouette of a notochord; the nucleus is red, the cytoplasm is light blue, and the mitochondrion is dark blue. Scale bar (also applicable to** d**–**o**) = 5 μm. **c** SBSEM image of block face showing cell *a1*, with fibers (Fi) in the cytoplasm and smooth lateral plasma membranes (arrowheads); the inset shows a mitochondrion. Scale bar = 1 μm. **d**–**o** Details of lamellar cells *a2–a13*, some with vacuoles (green). Inset in *a7* shows plasma membrane with microtendons (arrowheads) associated with the notochordal sheath. Inset scale bar = 1 μm

Figure 3a is an enlargement of the lamellar cells in zone *a*. From posterior to anterior these are designated *a1* through *a13*. No intercellular spaces intervene between lamellar cells in this zone (or anywhere else along the length of notochord reconstructed here). A more detailed view of cell *a1* is shown in Fig. 3b: at left in side view, and at right in posterior view against a light green silhouette of the notochordal cross section. The nucleus is indicated in red, the single mitochondrion in dark blue, and the cytoplasm in light blue. The cell has assumed the shape of a flattened plate, contrasting sharply with the lentoid and oval shapes of the other notochordal cells. A SBSEM image of cell *a1* (Fig. 3c) shows the cytoplasmic muscle fibers characteristic of lamellar cells; elsewhere the cytoplasm is strikingly lucent. In this newly formed lamellar cell, which spans almost the whole width of the notochord, the smooth plasma membrane lacks specialized structures associating it with the surrounding collagenous sheath of the organ.

Each frame in Fig. 3d–o shows a lamellar cell in side view (its narrow aspect) and in posterior view (its expanded, plate-like aspect). The cells in the series posterior to *a6* appear relatively undifferentiated. Although the cytoplasm is fibrous, no cytoplasmic vacuoles are present, and there are no specialized junctions associating the plasma membrane with the notochordal sheath. It is likely that some of these differentiating lamellar cells arise directly from newly produced posterior Müller cells deep in the notochord, while others originate from superficial Müller cells migrating inward from the dorsal or ventral surfaces of the organ. In contrast to the less differentiated lamellar cells, *a7–a12* contain a cytoplasmic vacuole (in green) varying in extent and dilation from one cell to the next, but always contacting the nucleus at some point. It is likely that these large vacuoles are derived from lysosomes, as is known to happen in vacuolated cells of the teleost notochord [21]. For adult lancelets, it has been assumed that the vacuoles in the lamellar cells play important roles in the biomechanics and extension of the notochord as a whole [19, 22].

The more advanced lamellar cells in zone *a* are associated with the notochordal sheath by numerous digitiform invaginations in the plasma membrane that we will call microtendons (Fig. 3i, inset, arrowheads). Although most of the lamellar cells in this zone are arranged in approximate order of increasing differentiation from posterior to anterior, cell *a13* appears much less differentiated than *a12*. This lack of order results because Müller cells can apparently intrude from the surface and differentiate into lamellar cells at any point along the entire anterior–posterior axis of the notochord. As a result, recently inserted lamellar cells can be found slotted in between more highly differentiated ones. In zone *a* lamellar cells, the mitochondria are spheres about 0.5 to 1 μm in diameter (Fig. 3c, inset), and each cell typically contains only one such organelle, rarely two or three.

### Zone *b*: lamellar cells

**Fig. 4:**
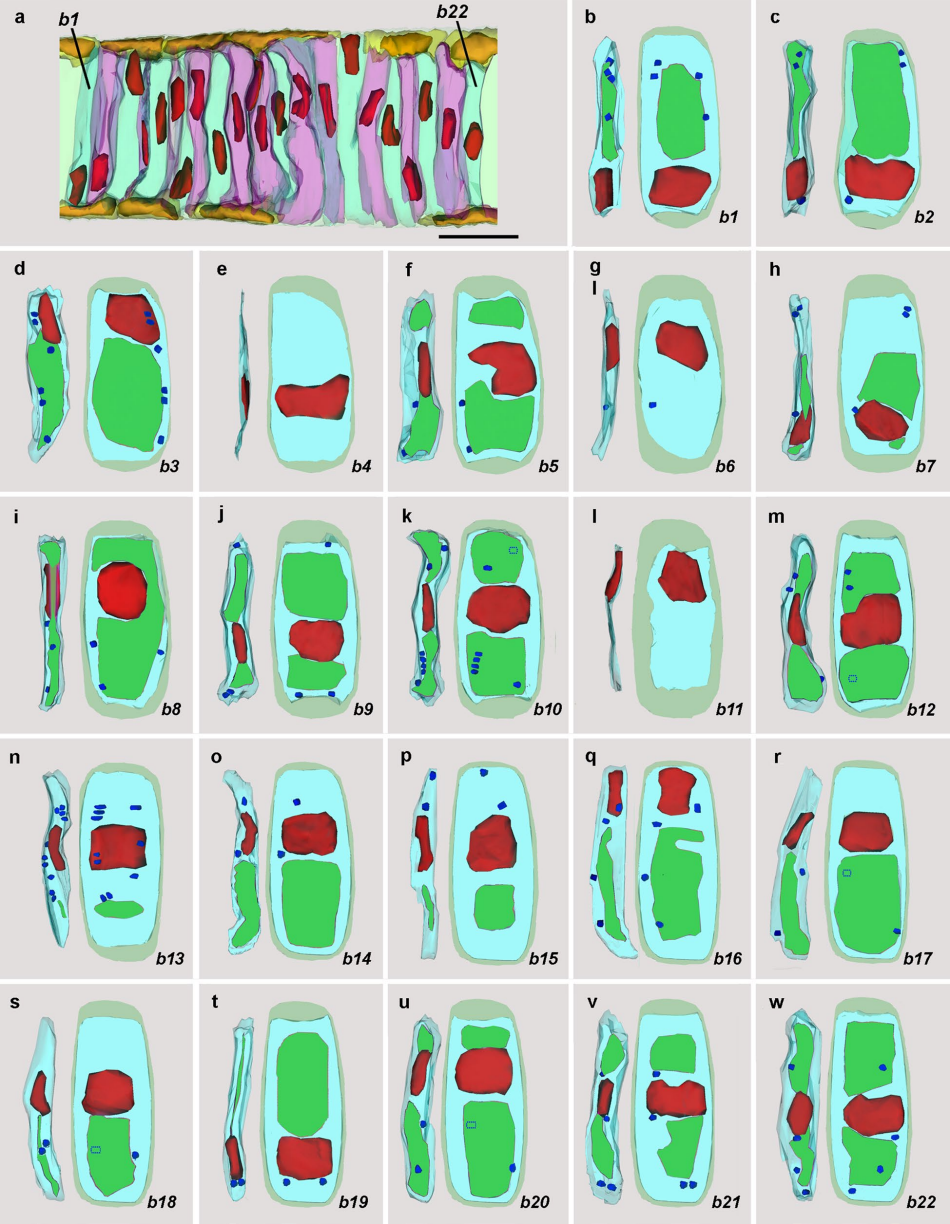
**a** Enlargement of zone *b* showing lamellar cells (with alternatively blue-green and pink cytoplasm) and Müller cells (light yellow cytoplasm). The lamellar cells from left to right, are designated *b1* through *b22*. **b**–**w** Details of lamellar cells *b1–b22*; orientation and color codes are as in Fig. 3; mitochondria hidden by intervening structures are indicated by dotted outlines. Scale bar in ** a **(also applicable to** c**–**w**) = 10 μm

The lamellar cells in zone *b* (Fig. 4a) are shown in detail in Fig. 4b–w and designated *b1* through *b22*. Most contain one or two large cytoplasmic vacuoles, and the number of mitochondria per cell ranges from 0 to 10, averaging about 3. Each mitochondrion is spherical to slightly oblong (the mean width and length, respectively, are 1.0 μm and 1.5 μm).

**Fig. 5:**
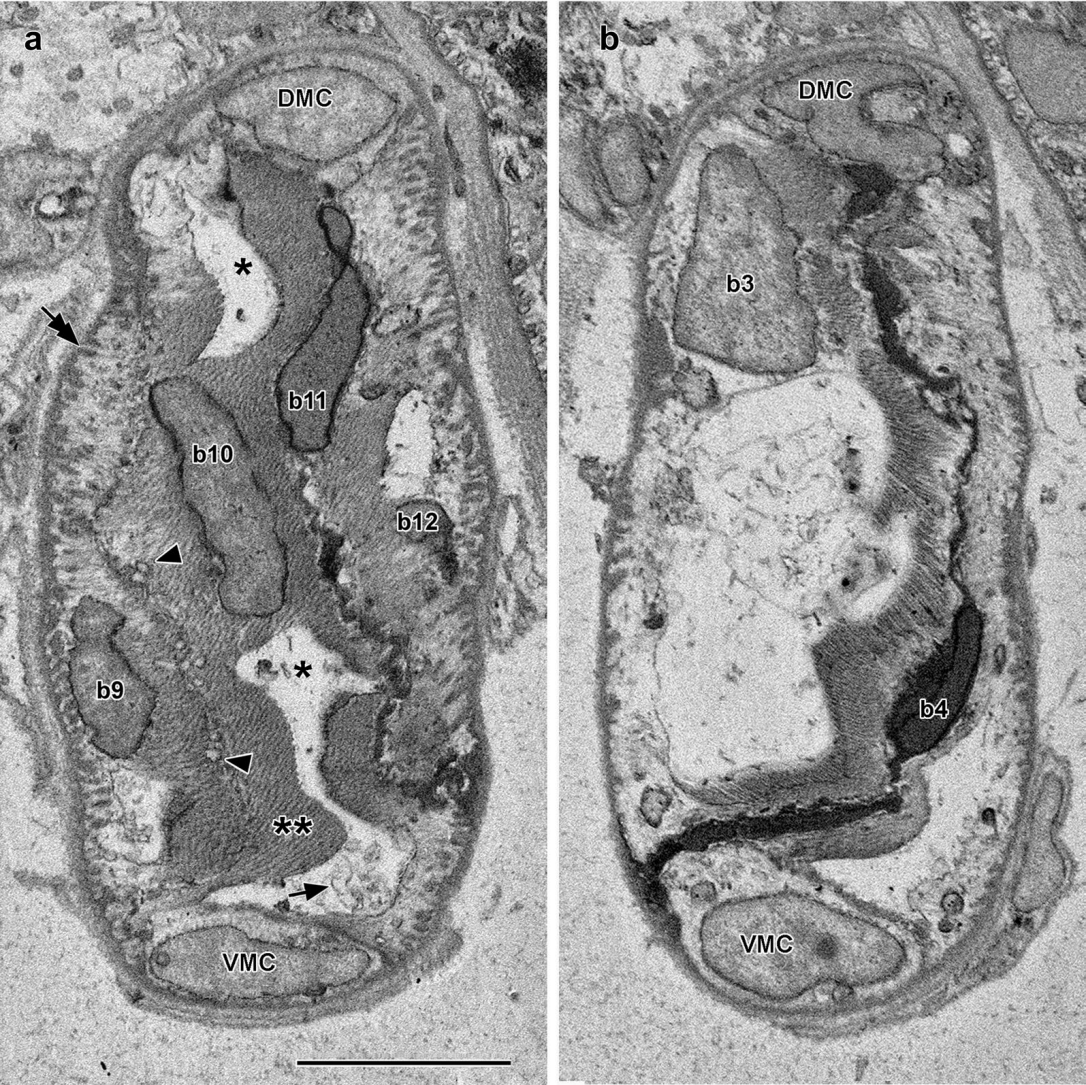
**a** SBSEM image of block face showing parts of lamellar cells *b9* through *b12* (labeled on the nuclei). For *b10*, asterisks indicate vacuoles dorsal and ventral to the nucleus, and arrowheads indicate sarcoplasmic reticulum vesicles. DMC and VMC are, respectively, dorsal and ventral Müller cells. Scale bar (also applicable to** b**) = 5 μm. **b** SBSEM image of block face showing parts of lamellar cells *b3* and *b4* (labeled on the nucleus). Abbreviations as in (**a**)

The lamellar cells are tilted at a slight angle to the plane of the blockface so that parts of several of them are visible in each SBSEM image. Figure 5a shows parts of four lamellar cells. Muscle fibers (twin asterisks) now predominate in the cytoplasm, and the vacuole lumens (single asterisks) contain some membranous profiles (single arrow) apparently originating from the inner surface of the organelle. The microtendons (tandem arrows) associating the lateral plasma membranes with the notochordal sheath are more abundant. In addition, just beneath the plasma membranes, there are a few vesicles of sarcoplasmic reticulum (Fig. 5a, arrowheads), presumably involved in muscle fiber contraction. In Fig. 4, there are three cells without cytoplasmic vacuoles. Cell *b6* has one mitochondrion in the fibrous cytoplasm and appears to be at a very early stage of differentiation. In contrast, cells *b4* and *b11* are clearly degenerating. Mitochondria are undetectable, and the cytoplasm is very dense and shrunken (Fig. 5b).

A few of the lamellar cells in zone *b* (and also more anteriorly) have the nucleus positioned at or near the surface of the notochord (overview in Fig. 1c; cells *b3* and *b16* in Fig. 4). Evidently, a Müller cell at the surface of the notochord can sometimes protrude its cytoplasmic region into the center of the organ and differentiate into a lamellar cell while the nucleus remains in a superficial position. It is likely that the nucleus eventually migrates inward to a more typical position, approximately in the center of the cell.

### Zone c: lamellar cells

**Fig. 6:**
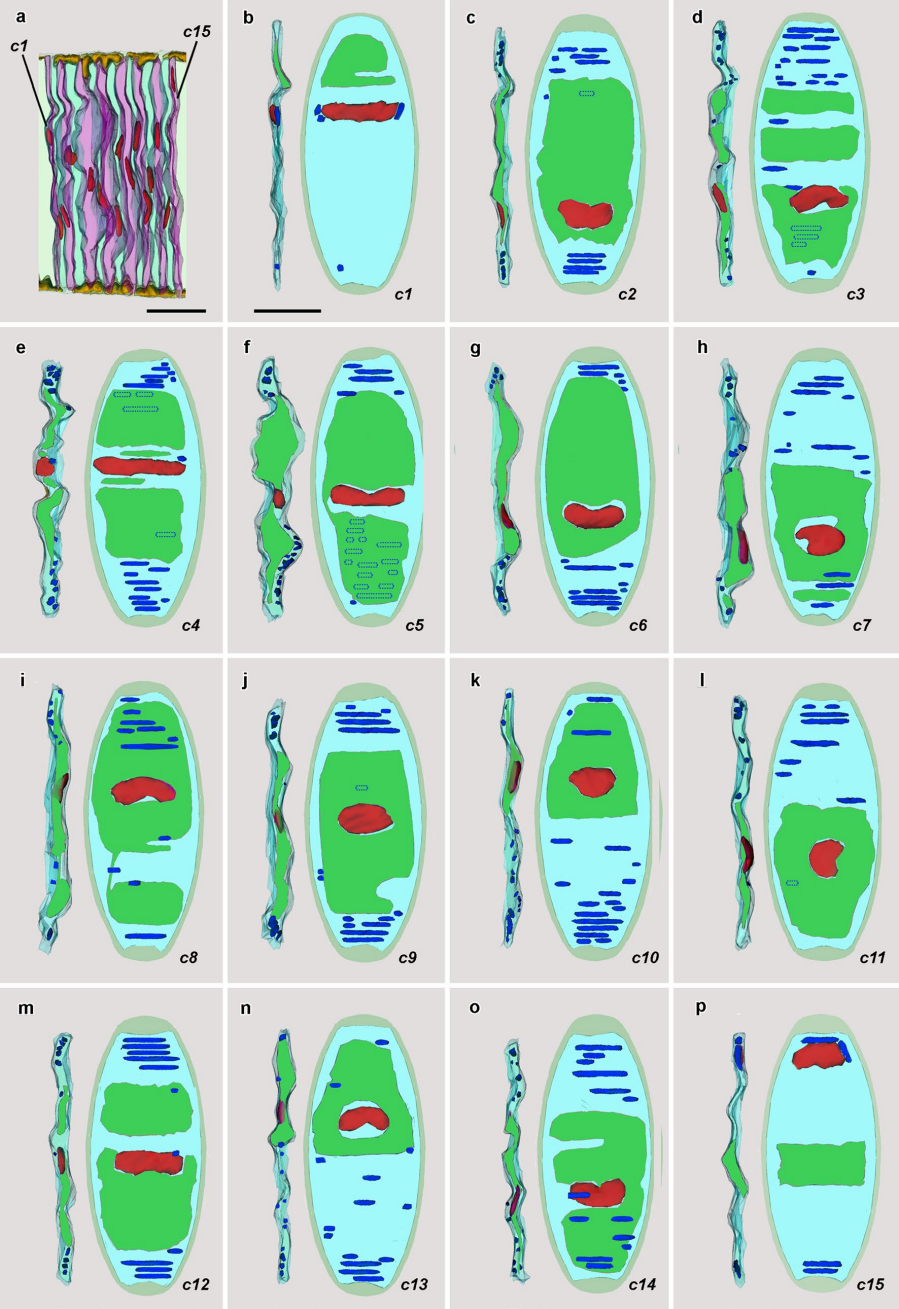
**a** Enlargement of zone *c* showing lamellar cells (with alternatively blue-green and pink cytoplasm) and Müller cells (light yellow cytoplasm); lamellar cells from left to right, designated *c1* through *c15*. Scale bar = 10 μm. **b**–**p** Details of lamellar cells *c1–c15*. Orientation and color codes same as in Fig. 3; mitochondria hidden by intervening structures are indicated by dotted outlines. Scale bar in **(b)** (also applicable to **c**–**p**) = 10 μm

**Fig. 7:**
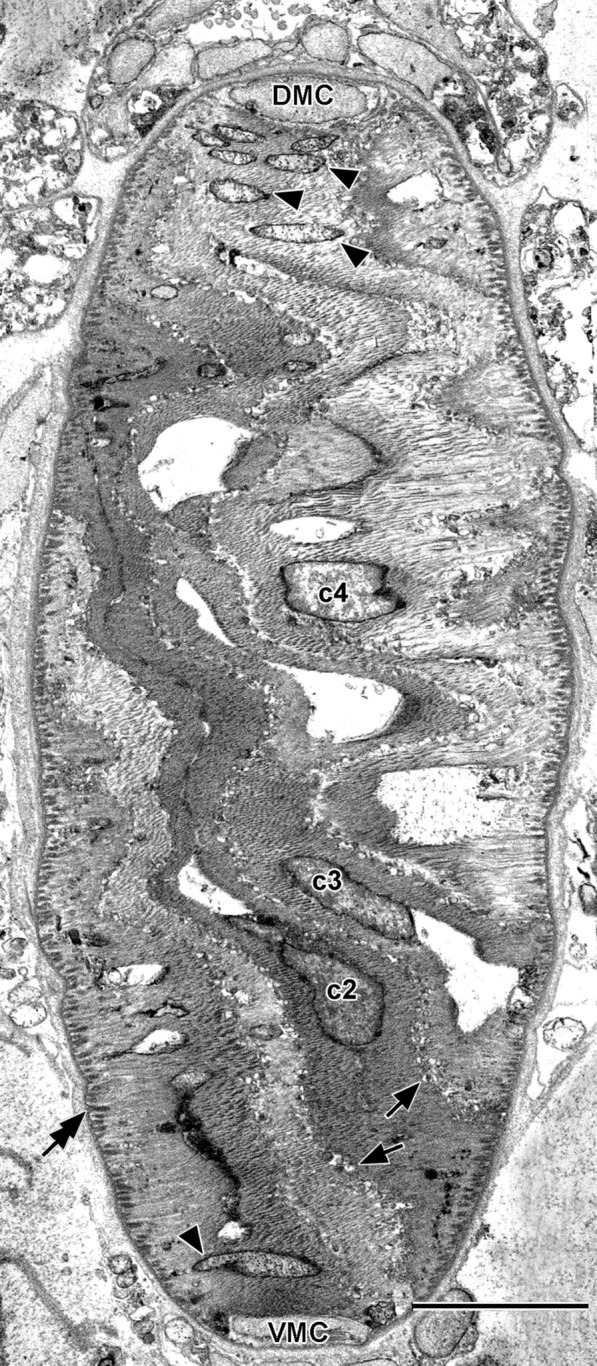
SBSEM image of block face showing parts of five lamellar cells. Arrows indicate sarcoplasmic reticulum vesicles, and arrowheads indicate mitochondria. DMC and VMC are, respectively, dorsal and ventral Müller cells. Scale bar = 5 μm

Figure 6a shows the structure of the lamellar cells in zone *c*. In Fig. 6b–p, they are illustrated in detail and designated, respectively, *c1* through *c15*. Each is characterized by 1 to 4 vacuoles that are often, but not invariably, closely associated with the nucleus. In comparison to the lamellar cells of zones *a* and *b*, those of zone *c* have more abundant vesicles of sarcoplasmic reticulum just beneath the plasma membrane (Fig. 7, single arrows), and the microtendons associating the lateral plasma membranes with the notochordal sheath are more conspicuous (Fig. 7, tandem arrows).

### Mitochondria in lamellar cells

**Fig. 8:**
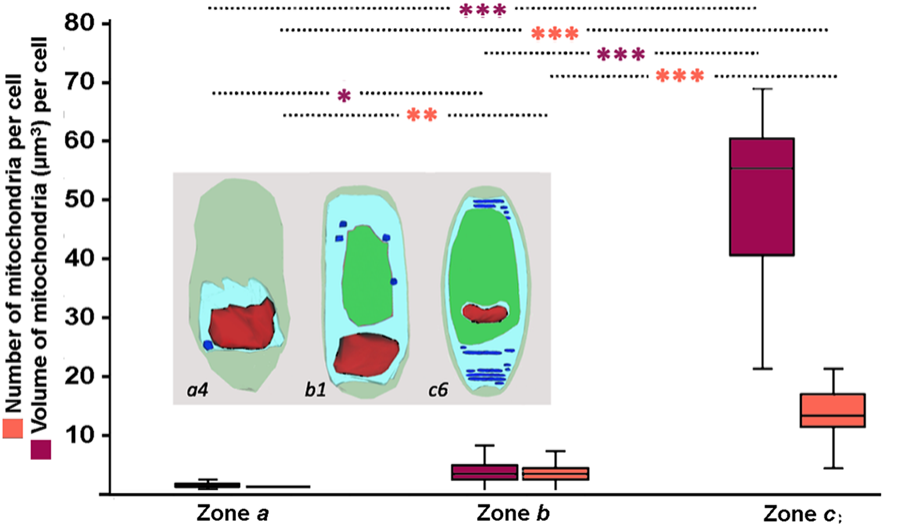
Mitochondrial number per cell and total mitochondrial volume per cell: comparison among lamellar cells from zones *a* (*n *= 13), *b* (*n* = 22), and *c* (*n* = 15) (representative examples in inset), Pairwise comparisons using the two-tailed Mann–Whitney indicate a significant difference across all three zones for both mitochondrial size and volume, with particularly numerous and long mitochondria in the zone *c* cells. *p < 0.02, **p < 0.0005, ***p < 0.0001

A striking feature of the lamellar cells in zone *c*, in comparison to those in zones *a* and *b*, is the marked increase in the number and length of the mitochondria (Fig. 7, arrowheads). Each mitochondrion in zone *c* lamellar cells is elongate, with a width of about 1 μm and a length ranging from 2 to 10 μm (mean 3.1 μm, standard deviation 2.2, *n* = 15). The mitochondrial number per cell ranges from 4 to 22 (mean 13.1, standard deviation 5.04, *n* = 15). There is in fact a significant difference in the number of mitochondria in lamellar cells in all three zones in pairwise comparisons (Fig. 8) (two-tailed Mann–Whitney test: *a* vs *b*,* U*(34) = 50.5, *Z* = 3.14, *p* = 0.0168; *b* vs *c*, U(36) = 11, Z–4.75,* p *< 0.00001; *a* vs *c*,* U*(27) = 0, *Z* = 4.47, *p* > 0.00001). Similarly, the mitochondrial volume per cell differed significantly among all three zones (Fig. 8) (two-tailed Mann–Whitey test: *a* vs *b*, U(34) = 40.5, *Z* = 3.48, *p* = 0.0005; *b* vs *c*, U(36) = 2, *Z* = − 5.03, *p* < 0.00001; *a* vs *c*,* U*(27) = 0, *Z* = 4.47, *p* < 0.00001). Although mitochondria were only visualized in lamellar cells at the tail end of the animal, it is likely that the volume of mitochondria per lamellar cell remains high along most of the length of the notochord before declining again near the anterior end of the organ.

The larger mitochondrial volume per cell in zone *c* might be correlated with a more advanced state of differentiation, as proposed for mammalian gut cells [23] and/or might reflect the higher energy needs or the muscular lamellar cells in more mature parts of the notochord. The mitochondria in the zone *c* lamellar cells have two additional features of unknown significance: first is their general tendency to occur in dorsal and ventral groups in the cytoplasm, and second is their occasional aggregation along the plasma membrane on either the anterior (*c5*) or the posterior (*c14*) side of the cell. Finally, two lamellar cells in zone *c* (*c1* and *c15*), evidently recently differentiated, included several mitochondria very closely associated with the nucleus. In general, such intimately juxtanuclear mitochondria are thought to be engaged in intensive molecular crosstalk with the nucleus to influence the early stages of differentiation [24, 25].

## Discussion

### The long-controversial structure of the lancelet notochord

Nineteenth century embryologists correctly understood that the embryonic lancelet notochord begins as a predominantly cellular structure [26]. However, with rare exceptions [6, 7], they mistakenly thought that the post-embryonic stages had notochords consisting chiefly of extracellular material, described as amorphous, plate-like, or fibrous. The correct view, that the core of the organ consists of nucleated lamellar cells, was not fully accepted until revealed by TEM in the twentieth century [8, 18, 27, 28]. Contemporaneously, it was also discovered that the fibrous component in the lamellar cell cytoplasm was a contractile apparatus rich in paramyosin (also called tropomyosin A) [29, 30]. In addition, TEM showed that dorsal extensions of lamellar cells in the trunk region receive neural input at synapses on the surface of the overlying CNS [8]. These features are unique to lancelets [31] and have caused some to question the notochord as a chordate homologue [32], although this is counterbalanced by other recent findings that support the homology [33–36].

### Intergeneric differences in lancelet notochords

The genera of cephalochordates comprise *Branchiostoma* and *Asymmetron* as well as the poorly known *Epigonichthys*. The details of notochord structure differ somewhat between *Branchiostoma* and *Asymmetron*. First, the Müller cells of *Branchiostoma*, as compared to *Asymmetron* [6, 8, 18] are: stellate, not oval; run in dorsal and ventral rows several abreast, not single-file; run in two additional rows, one in each dorsolateral position; are surrounded by voluminous fluid-filled spaces; and include some filament bundles and a conspicuous Golgi complex. It is unclear if these differences are real or simply result from comparing the trunk of one species with the tail of another species. In addition, the lamellar cells in adults of *Branchiostoma*, as compared to *Asymmetron*, contain less conspicuous vacuoles and are often separated by intercellular spaces, although the latter might be artifacts [18]. Moreover, the synaptic associations already mentioned between the lamellar cells and the CNS of *Branchiostoma* [8] were not found in the tail of *Asymmetron*, possibly because such synapses are limited to the trunk region of lancelets to influence notochord biomechanics there. Finally, it would be interesting to determine if the fine-structural details of the posterior terminus of the notochord in the genus-specific caudal process of *Asymmetron* are comparable to those in the more muscular tail of *Branchiostoma*.

### Cell population dynamics in the lancelet notochord

The present results suggest that the notochord of adult cephalochordates comprises populations of likely stem cells, progenitor cells, and terminally differentiated cells. Each of these is distinguishable from the others by distinctive morphology. Moreover, unequivocal intermediate cells are found at the boundaries between cell populations. This clarity of structure contrasts with some vertebrate stem cells and progenitor cells that can be distinguished only by their different gene expression and immunochemistry [37]. Because of the substantial collagenous sheath, mentioned above as surrounding the entire notochord, the notochordal cell populations appear well isolated from other tissues. This relative isolation from the surrounding tissues is established by the late embryonic stage [38]. Therefore, it would not be surprising to find that the notochordal stem cells (if one assumes that their identity will ultimately be confirmed) are tissue-specific in cephalochordates.

At the posterior end of the lancelet notochord, the presumed stem cells probably shift their positions only gradually and only over short distances as they self-renew and/or differentiate. It is also likely that the lamellar cells, under conditions of normal growth, do not move around in the core of the notochord. In contrast, more uncertainty surrounds the movements of the Müller cells. Some might differentiate into lamellar cells while still in the core of the notochord (as suggested by the arrangement of the nuclei in Fig. 2a), but most evidently reach the surface of the organ to join the dorsal or ventral row of their counterparts. It is not known whether Müller cells are added to the surface rows only posteriorly or more haphazardly. It even remains possible that these cells are motile and able to change places with their neighbors. The superficial Müller cells of *Branchiostoma* can proliferate, as suggested for adults in the older literature and more recently indicated by nuclear incorporation of bromodeoxyuridine in larvae [39]. However, an important question, yet unanswered, is whether they are constrained to divide not more than a finite number of times before differentiating into a lamellar cell—and thus fit the definition of progenitor cells as opposed to stem cells [40]. This question needs to be addressed by studying the details of notochordal cell proliferation. Although counting mitotic figures by electron microscopy underestimates the abundance of dividing cells in somatic tissues of adult cephalochordates [41], proliferation markers like BrdU [39] and phosphohistone H3 [42] can provide more reliable results.

It would be especially interesting to follow the fate of the Müller cells during regeneration after tail removal. Those remaining in the stump of the notochord would presumably be the source of new lamellar cells to lengthen the regenerating organ. This possibility is strengthened by the finding of Joseph [43] that a localized injury in the lancelet notochord is followed by proliferation and internalization of nearby Müller cells to repair the damaged core (which he mistakenly thought consisted primarily of extracellular matrix). A study of cephalochordate tail regeneration [42] found that a mass of small cells accumulated just posterior to the differentiated lamellar cells in the regenerating notochord. It has yet to be clearly demonstrated that these small cells were proliferating Müller cells, but it would not be surprising if they were. It also seems likely that, towards the completion of notochord regeneration, some of these actively proliferating cells would reverse the course of differentiation to re-establish the lost stem cells, as is known to happen in some regenerating vertebrate tissues [44, 45].

The present study focused on the posterior end of the notochord. However, in cephalochordates, the organ extends all the way up to the anterior tip the body, where the histological organization is virtually a mirror image of the posterior end. In a study of cell proliferation in larval cephalochordates [39], a few dividing cells were found at either extremity of the notochord. Moreover, in adult cephalochordates, the anterior tip of the notochord, if removed, can regenerate [42]. Thus there is little doubt that the notochord can grow anteriorly as well as posteriorly. It could well be that the population dynamics of the notochord cells are very similar at either end of the organ.

### Evolution of notochordal cell population dynamics in the chordates

The present study suggests stem cells might account for notochord growth in an adult lancelet, the best available proxy for the ancestral chordate. It is, therefore, interesting to consider how this feature may have evolved in the two other main groups of chordates—the tunicates and the vertebrates.

### Notochordal stem cells in tunicates?

In ascidian tunicates, determinate cleavage [46] produces a larval notochord without the participation of stem cells, and the organ persists for only a day or two before being destroyed at larval metamorphosis. The small, transitory notochord in ascidians accords with their general tendency to simplify and even lose ancestral features [47].

In another tunicate group, the appendicularians, the situation is more interesting because the notochord cells originally form in the embryo by an invariant cell lineage, but then continue proliferating along the length of the organ in the early adult [48]. However, because the appendicularian life span is typically only about 1 week, notochord growth might not involve a sub-population of stem cells. Instead, it remains possible that the increase in cell number results from divisions of progenitor cells with a limited potential for proliferation or from direct entry of differentiated cells into the cell cycle, as happens when the mammalian liver grows by proliferation of differentiated hepatocytes [49].

### Notochordal stem cells in non-mammalian vertebrates?

This section is focused on possible stem cells in adult notochords and omits the initial establishment of the organ during early embryology—when it can sometimes be problematical where and how to apply the concept of stem cells [50]. Even so, some of the work on late larval stages of teleosts and amphibians is relevant here and will be covered.

In adults of agnathans [51, 52] and several basal clades of fishes [53, 54], the notochord remains unconstricted by encroaching skeletal elements. Histologically, the notochord in these species comprises a thin peripheral layer of non-vacuolated cortical cells (a name that we will use here instead of the several synonymous terms in the literature) surrounding vacuolated cells of the inner core. The cortical cells have been called “chordoblasts” to indicate that they are thought to differentiate into core cells [51–54]. However, these studies include no speculation that a population of stem cells might be present in addition. It is conceivable that future work will show that the cortical cells are a progenitor population derived from stem cells that have escaped detection so far—either at the posterior end or perhaps arranged in some other pattern in the notochord.

Vertebrates other than those mentioned above often partially replace the notochord with cartilage and/or bone by the adult stage, dividing the organ into a series of relicts. In adult elasmobranchs [55] and teleosts [56, 57], each notochordal remnant is a conspicuous pad of tissue housed in a cavity formed intervertebrally by the apposition of the concave ends of neighboring vertebrae. In adult elasmobranchs [55] and teleosts [57], each notochordal pad consists of a cortical layer of small cells proposed to be chordoblasts that differentiate into the deeper vacuolated cells. Neighboring intervertebral pads often remain interconnected—but only by a tenuous strand of tissue passing through a small canal in the calcified and/or boney vertebrae. An added complexity, the notochordal pads of teleosts can include voluminous intercellular spaces [57]; such spaces within epithelia and mesothelia are relatively uncommon, but a good example is the tunnel of Corti in the inner ear epithelium of mammals [58].

In larval teleosts, the still-unconstricted notochord consists of three cell populations: cortical cells covering the surface, vacuolated cells at the core, and discoid cells at the posterior end of the organ [59–61]. At first glance, the arrangement of these three cell populations suggests a similarity with the adult lancelet notochord. However, in normally developing teleost larvae, the discoid cells do not behave like stem cells but evidently differentiate into cortical and core cells without dividing and thus disappear shortly before the vertebrae begin forming. Also in larval teleost notochords, experimentally damaged core cells are replaced by ingression of some cortical cells, although the internalized cells only form a dense plug without differentiating into typical vacuolated cells [62].

To date, no stem cells have been demonstrated in notochordal tissue of adult elasmobranchs and teleosts. However, in many such fishes, overall body growth continues throughout adult life, and their component tissues grow correspondingly. It is known that the slow, continuous enlargement of some organs in adult fishes and other vertebrates is supported by stem cells [63, 64]. These findings strengthen the likelihood that stem cells are similarly involved in the growth of the intervertebral pads of teleosts.

In adult amphibians and reptiles (in the broad sense to include birds), the fate of the notochord varies considerably from one species to the next. It is only rarely unconstricted, in which case a layer of cortical cells is presumed to differentiate into vacuolated cells of the core [65, 66]. The unconstricted notochords in larval frog tails have a similar structure and a similar conversion of cortical to vacuolated cells [67]. Conversely, the notochord of some amphibians and reptiles can be totally destroyed at the adult stage [68, 69].

In contrast to the above extremes, notochordal tissue of many amphibians and reptiles survives into adulthood, but becomes constricted [70–72]. For example, some species maintain the notochordal tissue in intervertebral regions (where it still contributes to limited flexibility of the vertebral column). In these species, the notochordal tissue becomes discontinuous due to the insertion of a cartilaginous plug in the mid-region of each vertebra.

In adults of other species of amphibians and reptiles, the notochordal relicts are arranged in just the reverse pattern. The notochordal tissue disappears intervertebrally, but remains in the mid-region of each vertebra with no role in spinal column flexibility. In such species, adjacent vertebrae often articulate at a joint comprising a skeletal convex surface fitting into a shallow skeletal socket with an intervening zone of cartilage—either solid (a synchondrosis) or incorporating an inconspicuous synovial cavity [68]. In one lizard with mid-vertebral relicts of notochord, cell proliferation was demonstrated, not in the vacuolated notochord cells, but only in cartilage cells in neighboring tissues [73]. The implication seemed to be that any growth of the notochordal relicts would require importation of cells from nearby cartilaginous regions, as is discussed below for mammals.

### Notochordal stem cells in mammals?

The previous section on non-mammalian vertebrates emphasizes that their adult notochord tissues are structurally diverse and that little attention has been paid to their possible stem cell component. Quite the opposite can be said of adult mammalian notochords: their vestigial structure is relatively uniform from one species to the next, and they have been intensively studied by stem cell biologists.

The subdivision of the embryonic mammalian notochord into relicts by the interposition of cartilaginous and bony vertebrae was described accurately over a century ago [74]. Each remnant in adult mammals is the nucleus pulposus, which represents the inner core of an intervertebral disc. The nucleus pulposus is not invested by a layer of chordoblast cells, but instead is bounded rostrally and caudally by flat plates of hyaline cartilage and peripherally by a thick layer of fibrous cartilage (the annulus fibrosus). Histologically, the nucleus pulposus of young mammals consists of a relatively voluminous extracellular material with vacuolated cells sparsely embedded in it. In some mammalian species, this disc histology persists throughout life [75]. However, in other species, vacuolated cells predominate in youth, but, with age, disappear as more and more chondrocyte-like cells become mixed with them. In older humans, even the latter begin to disappear.

The degenerative change in the nucleus pulposus regions of the human spinal column is currently driving the search for stem cell-based rejuvenation of the tissue. The approaches may involve introducing extra-vertebral cells into the tissue [76] or stimulating the proliferation of stem cells and progenitor cells already present [77]. This second approach is the one of interest here. The presence of proliferating cells in intervertebral discs of adult mammals is controversial. On the one hand, tracing of labeled proliferating cells indicates that augmentation of cell number in the nucleus pulposus depends on import of chondrocytes from more peripheral tissues [78–80]. On the other hand, cell type-specific targeting in Cre-Lox mice indicates that endogenous cell proliferation occurs within the adult nucleus pulposus itself [81, 82].

## Conclusion

The morphological data presented here strongly suggest that the growth of the notochord of adult lancelets depends on stem cells that produce progenitor cells; the latter, in turn, give rise to the highly differentiated cells constituting the core of the organ. This sequence, if verified by dynamic tracer experiments and immunohistochemistry, would be an exceptionally clear example of stem cell-based growth in a slowly growing adult organ.

For chordates in general, almost all of the work on the cellular basis of notochordal growth has been done on mammals due to its clinical relevance for vertebral disc disease. As mentioned above, the notochordal tissue of post-fetal mammals is present only as vestigial nucleus pulposus regions. The reduced state of the mammalian notochord is secondarily derived and should not distract attention from the likely importance of endogenous stem cells in the notochords of many other chordates. Lancelets are the most basal chordates, and it is reasonable to assume that the cell population dynamics underpinning growth of their notochord is an ancestral feature that carried forward in evolution. Our present results prompt us to suggest that careful searches should show that tissue-specific stem cells are a dynamic component of the notochordal tissue in many adult vertebrates, although perhaps not in the nucleus pulposus of mammals.

## References

[1] Delsuc F, Tsagkogeorga G, Latrillot N, Philippe H (2008). Additional molecular support for the new chordate phylogeny. Genesis..

[2] Simakov O, Marletaz F, Yue JX, O’Connell B, Jenkins J, Brandt A, Calef R (2020). Deeply conserved synteny resolves events in vertebrate evolution. Nat Ecol Evol.

[3] Yue JX, Yu JK, Putnam NH, Holland LZ (2014). The transcriptome of an amphioxus, *Asymmetron lucayanum*, from the Bahamas: a window into chordate evolution. Genome Biol Evol.

[4] Holland ND, Holland LZ (2016). The ups and downs of amphioxus biology: a history. Int J Dev Biol.

[5] Annona G, Holland ND, D’Aniello S (2015). Evolution of the notochord. EvoDevo.

[6] Lwoff B (1891). Über Bau und Entwicklung der Chorda von Amphioxus. Mitth Zool Sta Neapel.

[7] Klaatsch H (1895). Beiträge zur vergleichenden Anatomie der Wirbelsäule. III. Zur Phylogenese der Chordascheiden und zur Geschichte der Umwandlungen der Chordastruktur. Morphol Jahrb..

[8] Flood PR (1975). Fine structure of the notochord of amphioxus. Symp Zool Soc Lond.

[9] Holland ND (2011). Spawning periodicity of the lancelet, *Asymmetron lucayanum* (Cephalochordata), in Bimini. Bahamas Ital J Zool.

[10] Deerinck TJ, Bushong EA, Lev-Ram V, Tsien RY, Ellisman MH (2010). Enhancing serial block-face scanning electron microscopy to enable high resolution 3-D nanohistology of cells and tissues. Micros Microanal.

[11] Wanner AA, Genoud C, Friedrich RW (2016). 3-dimensional electron microscopic imaging of the zebrafish olfactory bulb and dense reconstruction of neurons. Sci Data.

[12] Peddie CJ, Collinson LM (2014). Exploring the third dimension: Volume electron microscopy comes of age. Micron.

[13] Titze B, Genoud C (2016). Volume scanning electron microscopy for imaging biological ultrastructure. Biol Cell.

[14] Fiala JC (2005). *Reconstruct*: a free editor for serial section microscopy. J Micros.

[15] Borrett S, Hughes L (2016). Reporting methods for processing and analysis of data from serial block face scanning electron microscopy. J Micros.

[16] Holland ND, Somorjai IML (2020). The sensory peripheral nervous system in the tail of a cephalochordate studied by serial blockface scanning electron microscopy. J Comp Neurol.

[17] Müller W (1871). Ueber den Bau der Chorda dorsalis. Jena Z Med Naturwiss.

[18] Eakin RM, Westfall JA (1962). Fine structure of the notochord of amphioxus. J Cell Biol.

[19] Stach T (1999). The ontogeny of the notochord in *Branchiostoma lanceolatum*. Acta Zool Stockh.

[20] Lai AG, Aboobaker AA (2018). EvoRegen in animals: time to uncover deep conservation or convergence of adult stem cell evolution and regenerative process. Dev Biol.

[21] Ellis K, Bagwell J, Bagnat M (2013). Notochord vacuoles are lysosome-related organelles that function in axis and spine morphogenesis. J Cell Biol.

[22] Yasuoka Y (2020). Morphogenetic mechanisms forming the notochordal rod: the turgor pressure-sheath strength model. Dev Growth Differen.

[23] Pradal G, Berreur M, Pouyet B, Riet A (2000). Differentiation of mucous neck cells into parietal cells: a new concept of mitochondrial biogenesis. Biol Cell.

[24] Pozzi A, Plazzi F, Milani L, Ghiselli F, Passamonti M (2017). SmithRNAs: could mitochondria “bend” nuclear regulation?. Mol Biol Evol.

[25] Wiese M, Bannister AJ (2020). Two genomes, one cell: mitochondrial-nuclear coordination via epigenetic pathways. Mol Metab.

[26] Hatschek B (1893). The amphioxus and its development.

[27] Welsch U (1968). Über den Feinbau der Chorda dorsalis von *Branchiostoma lanceolatum*. Z Zellforsch Mik Anat.

[28] Ruppert EE, Harrison FW, Ruppert EE (1997). Cephalochordata (Acrania). Microscopic anatomy of invertebrates, Volume 15, Hemichordata, Chaetognatha, and the invertebrate chordates.

[29] Flood PR (1967). The notochord of amphioxus; a paramyosin catch-muscle?. J Ultrastr Res.

[30] Flood PR, Guthrie DM, Banks JR (1969). Paramyosin muscle in the notochord of amphioxus. Nature.

[31] Almazán A, Ferrández-Roldán A, Albalat R, Cañestro C (2019). Developmental atlas of appendicularian *Oikopleura dioica* actins provides new insights into the evolution of the notochord and the cardio-paraxial muscle in chordates. Dev Biol.

[32] Ruppert EE, Harrison FW, Ruppert EE (1997). Introduction: microscopic anatomy of the notochord, heterochrony, and chordate evolution. Microscopic anatomy of invertebrates, Volume 15, Hemichordata, Chaetognatha, and the invertebrate chordates.

[33] McMenamin MAS (2019). Cambrian chordates and vetulicolians. Geosciences.

[34] Kugler JE, Passamaneck YJ, Feldman TG, Beh J, Regnier TW, Di Gregorio A (2008). Evolution of vertebrate notochord genes in the ascidian *Ciona intestinalis*. Genesis..

[35] Holland PWH, Koschorz B, Holland LZ, Herrmann BG (1995). Conservation of *Brachyury* (T) genes in amphioxus and vertebrates: developmental and evolutionary implications. Development.

[36] Shimeld SM (1999). The evolution of dorsoventral pattern formation in the chordate neural tube. Amer Zool.

[37] Carrieri C, Comazzetto S, Grover A, Morgan M, Buness A, Nerlov C, O’Carroll D (2017). A transit-amplifying population underpins the efficient regenerative capacity of the testis. J Exp Med.

[38] Schubert M, Holland LZ, Stokes MD, Holland ND (2001). Three amphioxus *Wnt* genes (AmphiWnt3, AmphiWnt5, and AmphiWnt6) associated with the tail bud: the evolution of somitogenesis in chordates. Dev Biol.

[39] Holland ND, Holland LZ (2006). Stage- and tissue-specific patterns of cell division in embryonic and larval tissues of amphioxus during normal development. Evol Dev.

[40] Lajtha LG (1979). Stem cell concepts. Differentiation.

[41] Lacalli TC, Hou SF (1999). A reexamination of the epithelial sensory cells of amphioxus (*Branchiostoma*). Acta Zool Stockh.

[42] Somorjai IML, Somorjai RL, Garcia-Fernandez J, Escriva H (2012). Vertebrate-like regeneration in the invertebrate chordate amphioxus. Proc Natl Acad Sci USA.

[43] Joseph H (1895). Über das Achsenskelet des Amphioxus. Z Wiss Zool.

[44] Chatzeli L, Simons BD (2020). Tracing the dynamics of stem cell fate. Cold Spring Harb Perspect Biol.

[45] Leedham SJ (2020). Reserving the right to change the intestinal stem cell model. Cell Stem Cell.

[46] Jiang D, Smith WC (2007). Ascidian notochord morphogenesis. Dev Dyn.

[47] Holland LZ, Gibson-Brown JJ (2002). The *Ciona intestinalis* genome: when the constraints are off. BioEssays.

[48] Søviknes AM, Glover JC (2008). Continued growth and cell proliferation into adulthood in the notochord of the appendicularian *Oikopleura dioica*. Biol Bull.

[49] Post Y, Clevers H (2019). Defining adult stem cell function at its simplest: the ability to replace lost cells through mitosis. Cell Stem Cell.

[50] Kimelman D (2016). Tales of tails (and trunks): forming the posterior body in vertebrate embryos. Curr Topics Dev Biol.

[51] Welsch U, Chiba A, Honma Y, Jørgensen JM, Lomholt JP, Weber RE, Mate H (1998). The notochord. The biology of hagfishes.

[52] Schwarz W (1961). Elektronenmikroskopische Untersuchungen an den Chordazellen von *Petromyzon*. Z Zellforsch Mik Anat.

[53] Schmitz RJ (1998). Comparative ultrastructure of the cellular components of the unconstricted notochord in the sturgeon and the lungfish. J Morphol.

[54] Budgett JS (1902). On the structure of the larval *Polypterus*. Trans Zool Soc Lond.

[55] Restović I, Vukojević K, Saraga-Babić M, Bočina I (2016). Ultrastructural features of the dogfish *Scyliorhinus canicula* (Pisces: Scyliorhinidae) notochordal cells and the notochordal sheath. Ital J Zool.

[56] Nowroozi BN, Harper CJ, De Kegel B, Adriaens D, Brainerd EL (2012). Regional variation in morphology of vertebral centra and intervertebral joints in striped bass,* Morone saxatilis*. J Morphol.

[57] Kryvi H, Rusten I, Fjelldal PG, Nordvic K, Totland GK, Karlsen T, Wiig H, Long JH (2017). The notochord in Atlantic salmon (*Salmo salar* L.) undergoes profound morphological and mechanical changes during development. J Anat..

[58] Sato M, Leake PA, Hradek GT (1999). Postnatal development of the organ of Corti in cats: a light microscopic morphometric study. Hear Res.

[59] Grotmol S, Kryvi H, Keynes R, Krossøy C, Nordvik K, Totland GT (2006). Stepwise enforcement of the notochord and its intersection with the myoseptum: an evolutionary path leading to development of the vertebra?. J Anat.

[60] Dale RM, Topczewski J (2011). Identification of an evolutionarily conserved regulatory element of the zebrafish col2a1a gene. Dev Biol.

[61] Seleit A, Gross K, Onistschenko J, Woelk M, Arturo C, Centanin L (2020). Development and regeneration dynamics of the *Medaka* notochord. Dev Biol.

[62] Lopez-Baez JC, Simpson DJ, Forero LL, Zheng Z, Brunsdon H, Salzano A, Brombin A (2018). *Wilms Tumor 1b* defines a wound-specific sheath cell subpopulation associated with notochord repair. eLife..

[63] Sîrbulescu RF, Ilieç I, Meyer A, Zupank GKH (2017). Additive neurogenesis supported by multiple stem cell populations mediates adult spinal cord development: a spatiotemporal statistical mapping analysis in a teleost model of indeterminate growth. Dev Neurobiol.

[64] Amato MA, Arnault E, Perron M (2004). Retinal stem cells in vertebrates: parallels and divergences. Int J Dev Biol.

[65] Welsch U, Storch V (1971). Fine structural and enzymehistochemical observations on the notochord of *Ichthyophis glutinosus* and *Ichthyophis kohtaoensis*. (Gymnophiona, Amphibia). Z Zellforsch Mik Anat..

[66] Howes GB, Swinnerton HH (1901). On the development of the tuatara, *Sphenodon punctatus*; with remarks on the egg, on the hatching, and on the hatched young. Trans Roy Soc Lond.

[67] Platz F (2006). Structural and experimental investigations of the functional anatomy and the turgor of the notochord in the larval tail of anuran tadpoles. Ann Anat.

[68] Winchester L, Bellairs AA (1977). Aspects of vertebral development in lizards and snakes. J Zool Lond.

[69] Rashid DJ, Chapman SC, Larsson HCE, Organ CL, Bebin AG, Merzdorf CS, Bradley R, Horner JR (2014). From dinosaurs to birds: a tail of evolution. EvoDevo.

[70] Jonasson KA, Russell AP, Vickaryous MK (2012). Histology and histochemistry of the gekkotan notochord and their bearing on the development of notochordal cartilage. J Morphol.

[71] Gegenbaur C (1862). Untersuchungen zur vergleichenden Anatomie der Wirbelsäule bei Amphibien und Reptilien.

[72] Wake DB (1970). Aspects of vertebral evolution in the modern Amphibia. Forma Functio.

[73] Alibardi L (2016). Localization of proliferating cells in the inter-vertebral region of the developing and adult vertebrae of lizards in relation to growth and regeneration. Anat Rec.

[74] Williams LW (1908). The later development of the notochord in mammals. Amer J Anat.

[75] Urban JPG, Roberts S, Ralphs JR (2000). The nucleus of the intervertebral disc from development to degeneration. Integ Comp Biol.

[76] Sakai D, Mochida J, Shapiro I, Risbud M (2014). Use of stem cells for regeneration of the intervertebral disc. The intervertebral disc.

[77] Lyu FJ, Cheung KM, Zheng Z, Wang H, Sakai D, Leung VY (2019). IVD progenitor cells: a new horizon for understanding disc homeostasis and repair. Nat Rev Rheumatol.

[78] Kim KW, Lim TH, Kim JG, Jeong ST, Matsuda K, An HS (2003). The origin of chondrocytes in the nucleus pulposus and histologic findings associated with the transition of a notochordal nucleus pulposus to a fibrocartilaginous nucleus pulposus in intact rabbit intervertebral discs. Spine.

[79] Henriksson HB, Thornemo M, Karlsson C, Hägg O, Junevik K, Lindahl A, Brisby H (2009). Identification of cell proliferation zones, progenitor cells and a potential stem cell niche in the intervertebral disc region. Spine.

[80] Henriksson HB, Svala E, Skioldebrand E, Lindahl A, Brisby H (2012). Support that migrating progenitor cells from stem cell niches contribute to normal regeneration of the adult mammal intervertebral disc. Spine.

[81] McCann MR, Séguin CA, Dettman RW, Wessels A (2016). Notochord cells in intervertebral disc development and degradation. J Dev Biol.

[82] Zheng Y, Fu X, Liu Q, Guan S, Liu C, Xiu C, Gong T (2019). Characterization of Cre recombinase mouse lines enabling cell-type specific targeting of postnatal intervertebral discs. J Cell Physiol.

[83] Holland ND (1964). Cell proliferation in post-embryonic specimens of the purple sea urchin (*Strongylocentrotus purpuratus*) an autoradiographic study employing tritiated thymidine.

